# Peer Intervention to Link Overdose Survivors to Treatment (PILOT): Protocol for a Multisite, Randomized Controlled Trial Conducted Within the National Institute on Drug Abuse Clinical Trials Network

**DOI:** 10.2196/60277

**Published:** 2024-09-17

**Authors:** Carrie Papa, Erin A McClure, Jenna McCauley, Louise Haynes, Timothy Matheson, Richard Jones, Lindsey Jennings, Tricia Lawdahl, Ralph Ward, Kathleen Brady, Kelly Stephenson Barth

**Affiliations:** 1 Department of Psychiatry and Behavioral Sciences Medical University of South Carolina Charleston, SC United States; 2 Hollings Cancer Center Medical University of South Carolina Charleston, SC United States; 3 San Francisco Department of Public Health San Francisco, CA United States; 4 Heritage Health Solutions Coppell, TX United States; 5 Department of Emergency Medicine Medical University of South Carolina Charleston, SC United States; 6 Faces and Voices of Recovery - Upstate South Carolina Greenville, SC United States; 7 Department of Public Health Sciences Medical University of South Carolina Charleston, SC United States

**Keywords:** opioids, overdose, nonfatal overdose involving opioids, peer support specialist, harm reduction, emergency department

## Abstract

**Background:**

The increase in opioid-related overdoses has caused a decrease in average life expectancy, highlighting the need for effective interventions to reduce overdose risk and prevent subsequent overdoses. Peer support specialists (PSSs) offer an appealing strategy to engage overdose survivors and reduce overdose risk, but randomized controlled trials are needed to formalize peer-led interventions and evaluate their effectiveness.

**Objective:**

This National Institute on Drug Abuse Clinical Trials Network (CTN) study is a multisite, prospective, pilot randomized (1:1) controlled trial (CTN protocol 0107) that aims to evaluate the effectiveness of an emergency department (ED)–initiated, peer-delivered intervention tailored for opioid overdose survivors (Peer Intervention to Link Overdose survivors to Treatment [PILOT]), compared with treatment as usual (TAU).

**Methods:**

This study evaluates the effectiveness of the 6-month, PSS-led PILOT intervention compared with TAU on the primary outcome of reducing overdose risk behavior 6 months after enrollment. Adults (aged ≥18 years; N=150) with a recent opioid-related overdose were identified and approached in the ED. Participants were screened and enrolled, either in the ED or within 7 days of ED discharge at research offices or in the community and then asked to complete study visits at months 1, 3, 6 (end of intervention), and 7 (follow-up). Participants were enrolled at 3 study sites in the United States: Greenville, South Carolina; Youngstown, Ohio; and Everett, Washington. Participants randomized to the PILOT intervention received a 6-month, PSS-led intervention tailored to each participant’s goals to reduce their overdose risk behavior (eg, overdose harm reduction, housing, medical, and substance use treatment or recovery goals). Participants randomized to TAU received standard-of-care overdose materials, education, and services provided through the participating EDs. This paper describes the study protocol and procedures, explains the design and inclusion and exclusion decisions, and provides details of the peer-led PILOT intervention and supervision of PILOT PSSs.

**Results:**

Study enrollment opened in December 2021 and was closed in July 2023. A total of 150 participants across 3 sites were enrolled in the study, meeting the proposed sample size for the trial. Primary and secondary analyses are underway and expected to be published in early 2025.

**Conclusions:**

There is an urgent need to better understand the characteristics of overdose survivors presenting to the ED and for rigorous trials evaluating the effectiveness of PSS-led interventions on engaging overdose survivors and reducing overdose risk. Results from this pilot randomized controlled trial will provide a description of the characteristics of overdose survivors presenting to the ED; outline the implementation of PSS services research in ED settings, including PSS implementation of PSS supervision and activity tracking; and inform ED-initiated PSS-led overdose risk reduction interventions and future research to better understand the implementation and efficacy of these interventions.

**Trial Registration:**

ClinicalTrials.gov NCT05123027; https://clinicaltrials.gov/study/NCT05123027

**International Registered Report Identifier (IRRID):**

DERR1-10.2196/60277

## Introduction

### Background

An increase in overdose deaths globally [1] and in the United States have caused a decrease in average life expectancy [2]. One of the greatest risk factors for a fatal overdose is experiencing a nonfatal overdose involving opioids (NFOO) in the previous year. Indeed, 6% to 10% of individuals who experience an NFOO die in the following year [3-5]. Survivors of NFOOs most commonly die of another overdose (67%) [3], with the highest risk period being the month following NFOO [5]. Interventions that reduce the risk of a subsequent overdose among NFOO survivors would substantially impact premature mortality.

US emergency departments (EDs) treat nearly 140,000 nonfatal overdoses per year [4], providing a point of contact to engage at-risk patients [6,7]. Although EDs have had some success in implementing certain treatment strategies to reduce overdoses, such as initiating medications for opioid use disorder (MOUDs) [8], which decrease mortality among those with opioid use disorder (OUD) [9,10], NFOO survivors have generally low rates of treatment engagement [3,11,12] and low readiness for treatment [13]. Furthermore, substance use disorder (SUD) diagnoses extracted from the medical record show that only 47% of those experiencing an NFOO meet the diagnostic criteria for an SUD and only 27% meet the criteria for OUD [14]. Taken together, traditional SUD treatment approaches initiated or referred in the ED may have limited success and uptake among an appreciable proportion of patients with NFOO who remain at high risk for a subsequent overdose. Strategies are needed to intervene with NFOO survivors in the ED to reduce the risk of subsequent overdoses, regardless of SUD diagnosis or interest and readiness for treatment or recovery.

Acknowledging that the population with NFOO may be more difficult to engage through traditional medical approaches, work has been focused on developing and evaluating ED interventions that are led by peer support specialists (PSSs) with lived experience with substance use that aim to increase treatment engagement. Preliminary results and recent randomized controlled trials characterizing PSS interventions have been promising [15-22], although few programs to date have been rigorously evaluated with a generalizable, multisite sample and with a focus on harm reduction, rather than SUD treatment initiation. One overdose prevention program developed through Faces and Voices of Recovery in Greenville, South Carolina (Faces and Voices of Recovery Overdose Recovery Coaching Evaluation [FORCE]) has shown preliminary success. FORCE is a recovery program led by PSSs that trains peers in overdose risk reduction and initiates connection in the ED when an overdose survivor is identified. A recent study evaluated the FORCE model among individuals who were hospitalized [23] and found impressive rates of treatment engagement 6 months after discharge (84% vs 34% in the control condition). While that study showed promise for PSS-led recovery coaching, the FORCE intervention has not yet been evaluated among those presenting the ED with an NFOO.

### This Study

This paper describes a study protocol to evaluate a PSS-led intervention initiated in the ED among NFOO survivors conducted within the National Institute on Drug Abuse (NIDA) Clinical Trials Network (CTN). This study adapted the PSS-led FORCE model into the Peer Intervention to Link Overdose Survivors to Treatment (PILOT), with the goal of testing this intervention through a multisite, pilot randomized controlled trial conducted in 3 EDs across the United States. This paper details study procedures, design, the PILOT intervention, and implementation of the CTN-0107 PILOT clinical trial, using Standard Protocol Items: Recommendations for Interventional Trials reporting guidelines [24]. Enrollment of study participants commenced in December 2021 and concluded in June 2023, with a final sample of 150 participants being enrolled and randomized.

## Methods

### Study Design Overview

This 2-arm, multisite, pilot randomized controlled trial will evaluate the effectiveness of the 6-month, PSS-led PILOT intervention compared with treatment as usual (TAU) initiated in the ED on the frequency of self-reported overdose risk behaviors at 6 months (end of intervention). This trial (also referred to as PILOT) aimed to enroll 150 patients (aged >18 years) who experienced a recent NFOO from 3 ED sites in the United States. In addition to routine ED care at the time of admission, all ED sites had peer recovery support systems in place in their ED that served as part of TAU (refer to *Study Setting* subheading mentioned subsequently).

**Figure 1:**
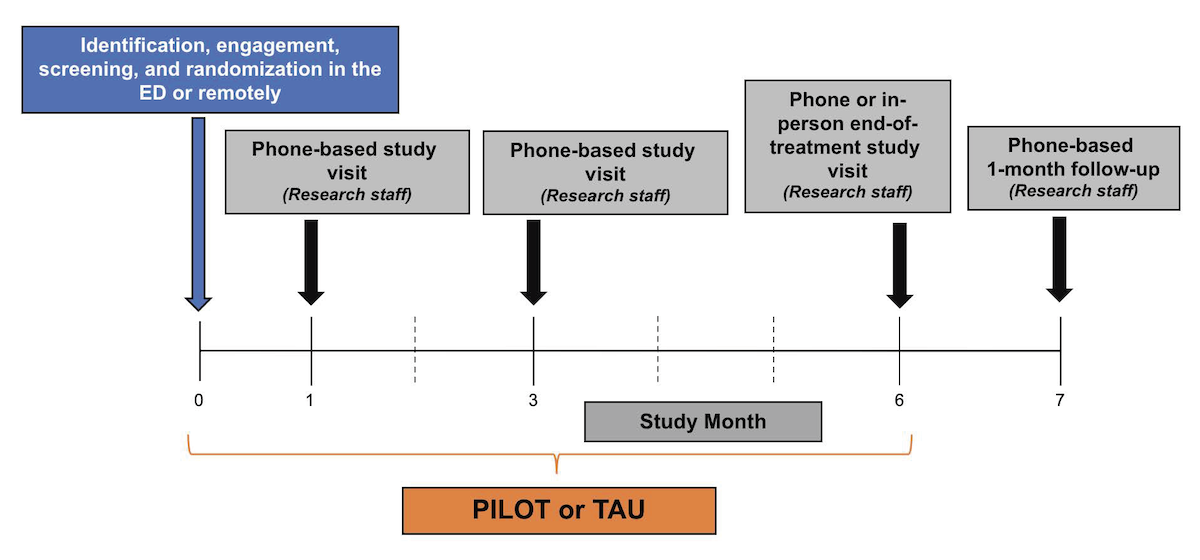
Overview of the CTN-0107 study design. CTN: Clinical Trials Network; ED: emergency department; PILOT: Peer Intervention to Link Overdose survivors to Treatment; TAU: treatment as usual.

Participants were approached during their ED admission or shortly after admission (Figure 1). If interested, eligible, and enrolled in the study, participants completed research study visits and data collection at months 1, 3, 6, and 7. Participants randomized to the PSS-led PILOT intervention engaged in the PILOT intervention for 6 months (refer to the description in the subsequent sections of the peer-led intervention). The primary outcome of this trial is a reduction in overdose risk behavior compared between participants in PILOT and TAU. Secondary outcomes include engagement in the study and intervention, as well as engagement in recovery and treatment, using a modified SUD Cascade of Care model (detailed description provided in the *Outcomes* section).

### Study Setting

CTN-0107 study sites were selected through a 3-phase process (Figure S1 in Multimedia Appendix 1) to determine the appropriateness for inclusion in this trial and the ability to recruit NFOO survivors in the ED. Sites with already functional ED–based peer recovery services in place were used, because the successful operationalization of a peer recovery team in an ED setting is a separate research question outside the scope and timing of this pilot study. Therefore, the TAU arm did include the possibility of interacting with a TAU ED–based PSS, and such interaction was documented. In addition, because MOUD receipt strongly predicts treatment retention, all sites were required to offer MOUD in the ED setting and to have a community system in place to provide MOUD for individuals without insurance. All sites were also required to be able to prescribe or dispense naloxone for overdose reversal.

The 3 sites selected for the study were Prisma Health—Upstate (Greenville, South Carolina), Mercy Health St Elizabeth Youngstown Hospital (Youngstown, Ohio), and Providence Regional Medical Center Everett (Everett, Washington). To optimize the fidelity of the intervention, all PSSs attended weekly group supervision with members of the PILOT intervention team (refer to the *Effective Supervision* section) and adhered to the PILOT manual if there were divergent peer recovery approaches.

### Ethical Considerations

The Medical University of South Carolina (MUSC) institutional review board (IRB) was the IRB of record for this study and reviewed and approved all study procedures, documentation, and safety events, etc. The study was approved by the IRB in February 2021 (Pro00103441). All study sites participating in this trial operated under the MUSC policies through a SmartIRB master reliance agreement. This trial is registered with ClinicalTrials.gov (NCT05123027).

### Approach, Screening, and Eligibility in the ED

After stabilization as part of routine TAU in the ED, research team members identified and approached patients who may have qualified for the study, in person or remotely (over the phone after ED discharge or if the patient leaves against medical advice but only if verbal permission to contact had been obtained). Routine TAU in the ED included medical care consistent with treating an overdose, including possible interaction with a TAU PSS, who offered Screening, Brief Intervention, and Referral to Treatment (SBIRT)–like services in the ED. If the participant was interested in the study and eligible (inclusion and exclusion criteria presented in Table 1), informed consent and screening procedures were performed either in the ED or up to 1 week following ED discharge. Participants were informed of the study components in greater detail, the required and optional assessments, potential risks and benefits of study participation, and the breakdown of study compensation (up to US $520). Patients could be admitted to the ED for any health issue, substance-related issue, or an NFOO. To be included, patients had to meet the criterion for an NFOO, including (1) being admitted to the ED for any reason and endorsed experiencing an NFOO within the past 72 hours or (2) being admitted to the ED with any substance-related condition and endorsed experiencing an NFOO in the past 30 days. These time frames and reasons for ED admission were meant to be inclusive to allow for non-NFOO reasons for ED admission while still capturing those with recent NFOO and who were at high risk of subsequent overdoses. This allowed for a more generalizable sample of patients in the ED with recent NFOOs but not necessarily presenting to the ED for an NFOO. Participants did not have to be computer or internet literate to be included in study procedures. Study sites were also able to provide study cell phones if a participant did not have their own device for contact and completing mobile phone study assessments.

**Table 1:** Study inclusion and exclusion criteria and rationale.

Criteria and description		Rationale
**Inclusion**		
	Aged ≥18 years	Study focused on adult population
	Meet 1 of the following *NFOO*^a^ criteria: (1) presented to ED^b^ for *any* reason in the past 48 hours and self-report having a known or suspected opioid-involved overdose in the past 72 hours and (2) presented in the ED in the past 48 hours for any SUD^c^-related issue and self-report having a known or suspected opioid-involved overdose in past 30 days	Definition of study sample
	Identify at least 2 additional contacts on the study locator information Form	Reduce loss to follow-up and to help ensure participant will provide useful data over the 7-month study
	English-speaking and able to provide written informed consent	Good clinical practice requirement to ensure informed consent
	Willing and able to confirm future SUD treatment receipt as evidenced by 2 out of 3 of the following: (1) signing appropriate releases for study staff to confirm treatment with follow-up provider, (2) having technology necessary to visualize medication bottles and transmit to study team using HIPAA^d^-compliant platform, and (3) able and willing to undergo toxicology tests	To help ensure participant will provide useful data and confirmation of treatment status
**Exclusion**		
	Identified as having had an intentional overdose as the index NFOO	Safety and outside of the scope of standard peer support
	Actively suicidal at the time of screening	Safety and outside of the scope of standard peer support
	Unable to complete study baseline procedures due to medical or psychiatric condition	Safety
	Meets the Office for Human Research Protection definition for prisoner status	Promote participant confidentiality and to help ensure participant will provide useful data
	Previously randomized as a participant in this study	To avoid confounding results
	Unwilling to follow study procedures	Safety and to help ensure participant will provide useful data

^a^NFOO: nonfatal overdose involving opioids.

^b^ED: emergency department.

^c^SUD: substance use disorder.

^d^HIPAA: Health Insurance Portability and Accountability Act.

Given the potentially short time that NFOO survivors were available to the research staff in the ED (given short ED patient disposition times), with verbal consent obtained during the ED visit, screening, consent, and baseline procedures were allowed to be completed within 1 week after ED discharge. All assessments and procedures conducted during screening, baseline, and during the study are shown in Table S1 in Multimedia Appendix 1. If patients were not interested in participating in the study or were not eligible, they were offered a brief survey assessing demographics, substance use, and overdose history and reasons for not being interested or eligible for the study.

### Randomization

Eligible participants were randomized 1:1 to the PILOT intervention condition or TAU. Explanation was provided to the participants that if they were randomized to the PILOT intervention, they would engage with a PILOT PSS with specialized training in overdose prevention (different than the TAU PSS in the ED unaffiliated with the study), and the PILOT PSS would coordinate with the TAU PSS during the ED visit to ensure no duplication of services and would engage with them for 6 months in the community. Participants randomized to TAU received all standard treatment services provided in the ED, and TAU PSSs conducted standard procedures as they would for any NFOO or patient with substance use admitted to the ED, with all sites offering an SBIRT-like model. The resources that TAU PSSs offered within the SBIRT-like model in their ED varied (and were reported through regular surveys completed by the site principal investigator and research staff), but all sites had the ability to offer naloxone as part of TAU, as required in site selection. PSS TAU interactions were generally contained within 1 meeting in the ED to provide treatment and harm reduction resources.

For participants randomized to the PILOT intervention, there was an on-call system for the PILOT PSSs such that they were available to come to the ED in person or remotely (over the phone or through research staff–facilitated video chat) to connect with participants. If possible, intervention participants began the PILOT intervention in the ED, immediately following randomization. If a connection was not made in the ED, the PILOT peer made attempts to contact the participant as soon as possible after randomization.

Randomization was stratified by study site and unstable housing status (yes or no). A permuted block randomization procedure with random block sizes was used to balance per site and housing status. The randomization schedule used balanced blocks of varying sizes within strata to ensure the lack of predictability along with relative equality of assignment across treatment groups. The randomization procedure was conducted centrally through the CTN Data and Statistics Center.

### Study Visits and Retention

During the 7-month study, participants were asked to complete study visits with the research staff at months 1, 3, 6, and 7. All research data were collected by the research staff at the study site and de-identified. PILOT PSSs did not collect research data or outcome data to ensure consistency and standardization across groups. PILOT intervention participants engaged with their assigned peers during the 6-month intervention. PILOT PSSs did not collect any data for research or outcome purposes, which is the responsibility of research coordinators for both PILOT and TAU groups. Assessments completed at study visits are shown in Table S1 in Multimedia Appendix 1, and all participants received weekly mobile diaries to complete during the study. To improve retention, study phones were provided to participants, as needed. Retention efforts were pursued throughout the study by research coordinators (for both PILOT and TAU participants) and PILOT PSSs (for PILOT-assigned participants only) followed up with their assigned PILOT participants throughout the 6-month intervention phase. If participants were unable to be reached by phone or could not attend study visits at research offices, assertive outreach was used by the research staff, which included mailed letters, text messages, visits in the community, and in rare circumstances, home visits with appropriate safety precautions in place, etc. Participants provided detailed locator information for at least 2 additional contacts if they could not be directly reached.

### PILOT Intervention Development, Peer Training, and Supervision

#### Involvement of People With Lived Experience in Protocol Development and Study Conduct

The value of inclusion of people with lived experience in SUD and recovery in developing and delivering this trial is recognized throughout study design, implementation, and dissemination of findings. Two PSSs (RJ and TL) have been integral members of the lead research team and intervention team since the inception of the study concept. These individuals bring years of lived experience in SUD recovery as well as supervision of PSSs within recovery-based organizations and have contributed meaningfully to the study design, implementation, and dissemination.

#### PILOT Intervention Development and Components

The PILOT intervention was adapted from the FORCE program, which used certified PSSs trained in engaging overdose survivors presenting to the ED. The CTN-0107 lead intervention team, including FORCE PSSs (RJ and TL), adapted the FORCE intervention (developed and implemented by RJ) and developed the PILOT intervention manual for this trial. The process of manual development included a literature search to complement what was learned from the FORCE implementation and an iterative process involving all members of the intervention team to identify the key principles of the intervention. The underlying philosophy of the intervention was grounded in a combination of motivational interviewing (MI), case management, health coaching, and assertive community engagement. The PILOT intervention manual was reviewed by an independent PSS, and feedback was incorporated before study enrollment began. The PILOT intervention manual was considered a living document to be further molded and adapted by the PSSs trained in the study.

The PILOT intervention manual describes three key principles as follows: (1) *assertive engagement,* (2) *participant-directed* care, and (3) *effective supervision*. *Assertive engagement* refers to the relationship between the PILOT PSS and the participant, as well as the expectation of active engagement with community partners. Characteristics of *assertive engagement* include (1) connection with and support of the participant informed by the PSSs’ lived experience, (2) the PSS taking responsibility for maintaining connection, (3) comprehensive knowledge of community resources, (4) linkage to services, (5) integration of family and other social supports, and (6) effective use of overdose harm reduction techniques.

The principle of *participant-directed* care in the PILOT intervention refers to the inclusion of participant-defined goals. Training on this approach, grounded in MI [25], was provided to PSSs during peer national training (described in subsequent sections) and is reinforced during regular supervision sessions. The PSS assumes responsibility for follow-up after ED discharge and continued engagement and contact throughout the intervention. By keeping in contact with the participants over time and using MI approaches, PSSs were available to individuals regardless of their level of interest in or readiness for treatment. Engagement with the individual was based on their needs, which evolved during the intervention and PSSs were encouraged to adapt their strategies, content, and services based on the individual participant.

*Effective supervision* was initiated from the time of PSS hire and continued throughout the study. At the study site level, supervision was conducted by a lead PSS, who was the on-site PILOT peer supervisor and provided at least weekly supervision. At the national level, a weekly, virtual supervision call for all PSSs was led by the intervention team, which included a PhD-level psychologist, a master’s level social worker, and 2 experienced PSSs. All PSS supervision sessions included informal discussions of participant cases, facilitated sharing of work-related experiences with feedback and support, and formal case presentations. National supervision also included a brief didactic, formal case presentations, and experiential review of key principles, including real-time practice of MI, promotion of self-care, awareness of safety in the field, and implementation of practical, participant-directed, and strengths-based case management.

#### PSS Training

All PILOT PSSs were required to hold a nationally recognized certified PSS certificate (or equivalent based on the state). PILOT PSSs participated in the virtual all-study national training led by the national intervention team and were required to complete all Human Subjects Protections and Good Clinical Practice training. The PSS training provided didactic and experiential (role-play) training using the PILOT intervention manual, including a discussion of PILOT PSS roles, responsibilities, and boundaries; detailed overviews of each treatment group; appropriate MI-based techniques and role-plays; and the importance of active supervision. During the national training, training on the MI approach was provided, and role-plays were conducted in groups of 2 PSSs with feedback provided by the intervention team. Postnational training was delivered via conference calls, webinars, and written materials. Competence in basic MI was reinforced through a taped recording of a mock participant session or a real-time role-play over videoconferencing, which was reviewed by the intervention team.

#### Peer Documentation and Fidelity

Given the interactions between a PSS and an individual are fluid, responsive to changing needs, and often spontaneous, the intervention team worked with the lead team PSSs to develop a checklist of the most common activities used (eg, outreach, engagement, discussion of lived experience, connection to resources, naloxone distribution, and identification of participant-directed goals). These activities were captured in the Peer Intervention Log that the PILOT PSSs filled out daily, describing the activities they used with a participant. The Peer Intervention Log was completed daily, even if no contact or intervention had been delivered on that day.

On the basis of feedback from the lead team PSSs, direct fidelity measurement (eg, recorded PSS-participant interactions) was found to be infeasible as this could inhibit spontaneity and rapport-building. Instead, metrics of PSS activity, content, frequency, etc, were collected during the 6-month intervention through the Peer Intervention Log. In addition, local and national supervisors performed fidelity assessments via case presentations during weekly supervision at the local level and during formal case presentations delivered by each PSS during national supervision.

### Measures and Assessments

#### Primary Outcome

The primary outcome measure for this study is a modified and expanded version of the Overdose Risk Behavior Checklist (ORBC), adapted from the study by Bohnert et al [26]. The modified ORBC (Figure S2 in Multimedia Appendix 1) was adapted from similar questionnaires to capture overdose risk behaviors in this or similar populations [17,26,27] (RELAY RCT; ClinicalTrials.gov identifier NCT04317053) and developed based on known factors associated with risk for overdose [26,28-33]. The PILOT ORBC is a 13-item scale, with 11 of the items used to generate a total risk score (ranging from 0-44); higher scores indicate greater frequency and number of overdose risk behaviors. The frequency of nonfatal and fatal overdoses will also be measured but given the relative infrequency of these events, the ORBC was a more proximal potential marker for change that could be measured within a 6-month pilot intervention.

#### Secondary Outcomes

The secondary outcomes of the study are (1) the number of steps achieved on a modified SUD Cascade of Care at 6 months after ED admission (Table 2); and (2) engagement with the study and PILOT intervention, measured by the number of potentially eligible patients *approached* in the ED compared with the number willing to be enrolled in study procedures and the length of enrollment in the trial among those randomized to PILOT (defined as the time from baseline to last meeting with the PILOT PSS). The SUD Cascade of Care was developed by the CTN-0107 lead team, informed by published work documenting an OUD Cascade of Care [34], to capture improvements that may otherwise be missed through existing assessments meant for those with SUDs or greater severity of substance use. The Cascade of Care outlines 10 steps representing different stages of SUD treatment or recovery engagement ranging from harm reduction to engagement in formal SUD treatment or MOUD. Participants do not need to meet the criteria for an SUD or OUD to be included in the steps achieved, which also includes steps toward overdose harm reduction and recovery capital. To assess steps achieved on the SUD Cascade of Care, participants are asked to complete an assessment battery, which includes a study-developed Harm Reduction Checklist, a Steps Achieved Assessment Form, MOUD Confirmation Assessment, a Diagnostic and Statistical Manual of Mental Disorders, 5th Edition Checklist for SUDs [35], a urine drug test, and the Assessment of Recovery Capital Scale [36] (refer to Table S2 in Multimedia Appendix 1 for the scoring rubric and Figure S2 in Multimedia Appendix 1 for locally developed assessments). All enrolled participants are considered to have 0 steps achieved at the time of the baseline visit, even if they are engaging in treatment or recovery services at the time of enrollment or baseline, with a maximum of 10 steps possible to be achieved by the end of treatment visit (month 6).

**Table 2:** Modified substance use disorder (SUD) Cascade of Care showing the cascade category, the steps achieved on the continuum of care, and the measure used to determine meeting that step.

Cascade and steps		Measure
**Overdose identification and harm reduction**		
	1. ↑ Harm reduction	Harm Reduction Checklist
**Engagement in care**		
	2. Any care	SAF 2-7^a^
	3. Regular care	SAF 2-7
**MOUD^b^ initiation**		
	4. Any MOUD	SAF 2-7
**MOUD retention**		
	5. MOUD × 1 month	SAF 2-7 and MOUD Confirmation Form
	6. MOUD × 3 months	SAF 2-7 and MOUD Confirmation Form
	7. MOUD × 6 months	SAF 2-7 and MOUD Confirmation Form
**Treatment response and remission**		
	8. ↓ SUD severity	*DSM-5*^c^ Checklist toxicology screen
	9. Early remission	*DSM-5* Checklist toxicology screen
	10. ↑ Recovery score	Assessment of Recovery Capital Scale

^a^SAF 2-7: Steps 2-7 Achieved Form.

^b^MOUD: medication for opioid use disorder.

^c^DSM-5: Diagnostic and Statistical Manual of Mental Disorders, Fifth Edition.

#### Safety Assessment

The population with NFOO is at high risk of adverse events and subsequent overdoses, and this study conducted targeted safety monitoring. Targeted safety monitoring events included participant deaths, overdoses, ED visits, and hospitalizations. Assessment for suicidality was screened for and handled according to approved safety procedures at all study visits. In addition, study staff and PSS safety protocols for community visits were developed when conducting in-person visits in the community. Safety and data oversight is provided by the NIDA CTN and Emmes Clinical Coordinating Center, site-level IRBs, and the MUSC IRB, as well as a Data Safety Monitoring Board that meets annually.

### Statistical Analysis Plan

#### Primary Outcome Analyses

The primary outcome is the effectiveness of PILOT (compared with TAU) as measured by the past month’s total score of the self-report ORBC assessment at month 6 (end of intervention). The study hypothesis is that PILOT intervention participants will have a lower ORBC total score at month 6 (ie, lower frequency of self-reported overdose risk behaviors) compared with TAU participants. The primary outcome of ORBC total score at month 6 will be analyzed with a longitudinal mixed effect Poisson regression model incorporating total score at earlier time points. Fixed effect covariates include baseline ORBC total score, treatment assignment, days in the study (time since randomization), site, stratum, and an interaction between treatment and days. Days will be treated as a categorical variable. A random effect will be included to account for repeated measures per participant. The treatment effect will be given as a rate ratio (RR; as the exponential of group and days interaction at month 6 plus the main effect of randomized group) along 2-sided *P* values and 95% CIs. This can be interpreted, conditionally, as a ratio of the mean risk behaviors for those assigned to PILOT divided by the mean risk behaviors for those assigned to TAU at month 6, while all other variables are the same.

#### Secondary Outcome Analyses

For the number of steps achieved along a modified SUD Cascade of Care, analysis will be similar to that for the primary outcome, with an overdispersed Poisson regression model with fixed effect covariates for treatment, site, and stratum. It is noteworthy that no baseline score covariate is included as all participants will be considered to have achieved 0 steps at baseline. An RR with 95% CI and *P* value will be reported. Participants entering the trial may not have a primary diagnosis of OUD or any SUD. Because of this, some steps may not be eligible at baseline. If, during the study, the participant endorses OUD or other SUD, they will become eligible for these steps. Alternatively, with the increasing co-use and contamination of methamphetamine with fentanyl, people with primary methamphetamine use disorder (and no diagnosis of OUD) may be placed on MOUD, and this study will measure that within this population.

Other secondary outcomes (ie, the number of participants approached, the number of participants who were willing to engage with PILOT peers, and the percentage of those approached who were willing to engage) will be reported by summary statistics with 95% CIs. The length of engagement with PILOT will be similarly summarized.

#### Power Analysis and Sample Size Estimation

Power analyses and sample size calculation were based on the primary outcome of comparing ORBC total score differences at month 6 between PILOT and TAU participants. The self-reported overdose risk behaviors to be used for this trial are modified and expanded from a version used by Bohnert et al [26], in which the maximum score was 32, with average baseline scores of 3.3 and 3.8 in their control and intervention groups, respectively. They used a Poisson regression model to estimate the intervention effect, reported to be a 0.72 RR. For the power simulations conducted for this study, a baseline mean score of 3.55 was used, with a treatment effect of 0.72. With a mean of 3.55 at baseline and an RR of 0.72, there is over 90% power to detect the expected treatment effect with a total sample size of 150.

#### Missing Data

The prespecified primary outcome analysis method considers all available data so that special provisions are not needed for missing data. However, missing data handling methods, such as multiple imputation, may be considered as possible sensitivity analyses. By definition, the secondary outcome of the number of steps achieved on the modified SUD cascade of care will have no missing data. Either evidence was previously obtained that a step was achieved, or evidence was never collected that the step was achieved (and thus was not achieved).

## Results

The overall goal of this pilot trial is to provide data on the potential effectiveness of a 6-month, specialized, PSS-led intervention for NFOO survivors presenting to ED settings in reducing overdose risk behavior at 6 months following ED admission. This study will also provide important information characterizing NFOO survivors presenting to the ED as well as the rate of engagement with and content of PSS-delivered services over the 6 months of intervention.

The PILOT study was effectively implemented at 3 geographically diverse sites in the United States, with enrollment of study participants commencing in December 2021 and concluding in June 2023, meeting the enrollment goal of 150 study participants. Final follow-up visits occurred through February 2024. As of July 2024, the PILOT study has developed and delivered an ED–based, PSS-led intervention within a research model and completed enrollment, demonstrating the feasibility of both completing a multisite trial in this area and recruiting the target population. Final primary and secondary outcome analyses within the final study report are expected in August 2024, with dissemination expected in October 2024.

## Discussion

### Overview

Outcomes of this trial will contribute important information to the field, including (1) a detailed description of the characteristics of and 6-month course of NFOO survivors presenting to 3 geographically different ED settings between December 2021 and June 2023; (2) a description of the nature, content, and dosing of a research-delivered PSS intervention; and (3) lessons learned in the research implementation of an ED-initiated PSS intervention. Study results will also contribute much-needed, rigorous, randomized, controlled data to the growing field of research and interventions to address overdoses and reduce the risk for subsequent overdoses among a generalizable sample of NFOO survivors.

### Design Decisions

Several key design decisions were made regarding the most appropriate sample for this study and outcome measurements. As a randomized pilot study, this protocol was designed to inform a larger, multisite randomized trial. Therefore, all procedures used in the trial attempt to test those procedures intended for the implementation in the subsequent larger trial [10].

The study enrolled patients in ED who had experienced a recent NFOO, though a diagnosis for OUD or SUD was not required for inclusion. Even among NFOO survivors who acknowledge SUD, treatment engagement is low and decreases with each additional month after overdose [14]. Moreover, given that less than half of the population with NFOO identifies as having SUD, this low level of treatment entry and decline in treatment use over time raises the critical question of what treatment or risk reduction measures are appropriate for NFOO survivors who do not identify as having an SUD and how to improve the retention of those with SUD who enter treatment after an overdose [21,22]. PILOT was designed to assess and address overdose risk factors for those who survive an NFOO, regardless of whether the individual identifies as having an SUD or is interested or ready for treatment entry. As it has also been shown that NFOO survivors without SUD or OUD are less likely to engage with outreach efforts [11], the primary outcome measure, screening, and PILOT intervention were tailored to include risk reduction for individuals in any SUD category. Specifically, the modified SUD Cascade of Care was developed to ensure several steps could be achieved by those without SUD or for those for whom MOUD is not indicated or desired. Although the traditional SUD Cascade of Care is used at a population level and not an individual level, for the purposes of this pilot study, the Cascade of Care logic was felt to be the most inclusive to identify where, outside of formal treatment engagement, PSS services might improve outcomes for NFOO survivors.

For overdose characteristics, the PILOT study included individuals in the ED who experienced an overdose in the past 30 days and considered an individual to have experienced an NFOO if they self-reported affirmatively that they believed they experienced an overdose and that the overdose *may have* involved opioids. These broader characteristics of overdose were chosen for several reasons. First, this approximates ED clinical practice of self-reported overdose. Second, urine toxicology cannot always identify very recent drug exposure, and some individuals admitted to the ED after an NFOO may not be confident which drugs were involved with the NFOO. Third, not all individuals who experience NFOOs receive naloxone or seek medical attention, even after naloxone is administered (therefore, naloxone administration or post-NFOO medical attention was not required and a 30-day window for overdose was allowed for those who presented to the ED with an SUD-related condition). If an individual denied having an overdose, even if they were given a reversal agent or it was medically assessed to be an NFOO, they were not eligible for inclusion, given the PILOT intervention specifically focuses on overdose risk behaviors. Design decisions regarding the PILOT study population aimed to maximize inclusivity, generalizability, and representation of the heterogeneity of the population with NFOO during the period of the opioid crisis this study was delivered.

Finally, our primary outcome was based on self-reported overdose risk behavior (via the ORBC) rather than subsequent overdoses between groups. Although the frequency of self-reported nonfatal overdoses and National Death Index-reported fatal overdoses Index will be measured, given the relative infrequency of overdose events, the ORBC was considered a more proximal potential marker for change that could be measured within a 6-month pilot intervention. The ORBC was developed based on factors associated with overdose, and the relationship between ORBC scores and overdose events will be an exploratory analysis in this study.

### Limitations

This study protocol and design include several limitations. The recruitment and enrollment of study participants in the ED following an overdose is a logistically challenging time to engage potential participants, especially when asking individuals in the ED for an acute medical event to complete extensive screening and baseline procedures. Similarly, informed consent during the ED visit presents a challenge for the research staff to ensure that participants are truly informed regarding study procedures. Through extensive staff training and oversight, we ensured that participants consenting to the study understood study procedures, risks, potential benefits, etc and were able to provide informed consent. These measures may have favored those with a higher level of functioning and cognitive ability required to understand informed consent and excluded those who experienced a very recent overdose who may not have been cognitively, physically, or emotionally well enough to complete informed consent. To mitigate this limitation, the study design incorporated the option to obtain verbal consent to follow up with a potential participant after they were home and feeling better. We also acknowledge the potentially important variables that we did not collect in this study to keep the assessment battery as minimal as possible. For example, the study did not evaluate the cost-effectiveness and other key variables that may affect substance use and craving, such as hormonal changes due to the menstrual cycle in biological women. However, analyses will evaluate outcomes by gender, age, and race and ongoing studies are evaluating the cost-effectiveness of PSS services. Finally, although this study measured nonfatal and fatal overdoses as exploratory measures, we acknowledge the choice of a self-report measure (eg, ORBC) as the primary outcome measure for PILOT is a limitation. Data from this pilot study will inform and power future studies to more rigorously determine the efficacy of the peer-led PILOT intervention on overdoses in this population at high risk.

### Conclusions

This PILOT study will evaluate the preliminary effectiveness of a 6-month, PSS-delivered intervention on reducing self-reported overdose risk behaviors among 150 individuals in the ED who experienced a recent NFOO. PILOT will contribute much-needed, rigorous, randomized, controlled data to a growing field of research on interventions aimed at preventing overdoses and reducing overdose risk among a generalizable sample of NFOO survivors. In addition to describing and providing preliminary effectiveness data for a PSS-led intervention targeting overdose risk behaviors, this study will also describe the characteristics of the NFOO population from December 2021 to June 2023, at the height of the overdose crisis, which will inform intervention targets for this population at high risk. PSS services are already being incorporated into medical settings to advocate for and address the needs of populations with SUD at high risk, and PILOT results will aid in implementing the most effective PSS practices for and inform a larger study powered to measure overdose events (fatal and nonfatal). Future work in this area is needed for the implementation of PSS services in EDs and other medical settings, as well as economic analyses to demonstrate the potential cost savings of implementing PSS services.

## Appendix

Multimedia Appendix 1 Supplementary tables and figures.

## Abbreviations

CTN: Clinical Trials Network
ED: emergency department
FORCE: Faces and Voices of Recovery Overdose Recovery Coaching Evaluation
IRB: institutional review board
MI: motivational interviewing
MOUD: medication for opioid use disorder
MUSC: Medical University of South Carolina
NFOO: nonfatal overdose involving opioids
NIDA: National Institute on Drug Abuse
ORBC: Overdose Risk Behavior Checklist
OUD: opioid use disorder
PILOT: Peer Intervention to Link Overdose survivors to Treatment
PSS: peer support specialist
RR: rate ratio
SBIRT: Screening, Brief Intervention, and Referral to Treatment
SUD: substance use disorder
TAU: treatment as usual

## Data Availability

Data sets that will be generated during and analyzed during this study will be made available on the NIDA Clinical Trials Network data share website. Any questions regarding data or availability can be sent to the lead investigator KB.
