# Supplementary material for: Peer Intervention to Link Overdose Survivors to Treatment (PILOT): Protocol for a Multisite, Randomized Controlled Trial Conducted Within the National Institute on Drug Abuse Clinical Trials Network
Source: JMIR Res Protoc. 2024 Sep 17;13:e60277. doi: 10.2196/60277 (PMC11445628; doi:10.2196/60277)
Supplement: Multimedia Appendix 1 [file resprot_v13i1e60277_app1.pdf]

Figure S1. Flow diagram of the site selection process and criteria used to determine study sites for inclusion in PILOT.

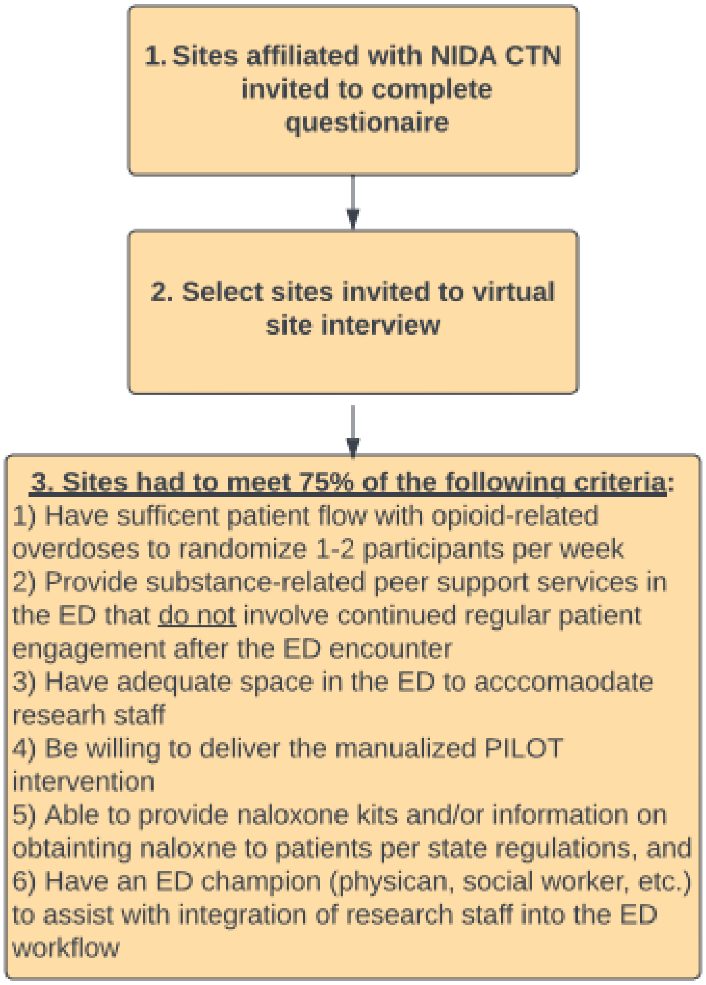

Figure S2. CTN-0107 Assessments for primary and secondary outcomes, including: 1) the Modified Opioid Risk Behavior Checklist (ORBC), and 2) Lead Team-developed assessments for the secondary outcome measure (SUD Cascade of Care), including the Harm Reduction Checklist (HRC), Steps Achieved Form (SAA) and MOUD Confirmation Assessment.

Opioid Risk Behavior Checklist (ORBC)

Administered at all study visits by RA

RA SCRIPT: *The next questions are about your use of illicit opioids (like heroin or fentanyl) and opioid pain medications in the past month. We are interested in your use of opioids that have been prescribed to you by a doctor as well as opioid medications or illicit opioids that you got from someone other than a doctor. For these questions, please think about the* ***past month****.*

	Never	Rarely	Sometimes	Often	Very Often	Refused
1. In the past month, how often have you used illicit opioids (like heroin or fentanyl) or opioid pain medications **when nobody else was around?**						
2. In the past month, how often have you used illicit opioids (like heroin or fentanyl) or opioid pain medications in a **place where you don't usually use them** (in a place that you had NEVER used in before, such as a different house or apartment, or a different public space)?						
3. In the past month, how often did you **drink alcohol within 2 hours** before or after using illicit opioids (like heroin or fentanyl) or opioid pain medications?						
4. In the past month, how often did you **take sedatives (such as Xanax or Valium) within 2 hours** before or after using illicit opioids (like heroin or fentanyl) or opioid pain medications?						
5. In the past month, how often did you use illicit **opioids (like heroin or fentanyl) or opioid pain medications within 2 hours** of one another?						
6. In the past month, how often did you **use crack or cocaine within 2 hours** before or after using illicit opioids (like heroin or fentanyl) or opioid pain medications?						
7. In the past month, how often did you **use crystal/meth within 2 hours** before or after using illicit opioids (like heroin or fentanyl) or opioid pain medications?						
8. In the past month, how often have you **increased the amount** of illicit opioids (like heroin or fentanyl) or opioid pain medications you used to more than you usually use?						
9. In the past month, how often have you used illicit opioids (like heroin or fentanyl) or opioid pain medications **behind a locked door?**						
10. In the past month, how often have you **snorted** any drugs?						
10a. [If ORB10 1,2,3 or 4]. In the past month, did you **switch from snorting** to other ways of using	No	Yes	Refused			

drugs (like injecting, smoking, or swallowing) for 7 days or longer, for any reason?						
10b. [If ORB10 1,2,3 or 4]. In the <u>past month</u>, did you ever **snort fentanyl** (intentionally or accidentally)?	No	Yes	Not Sure	Refused		
11. In the <u>past month</u>, how often have you **injected** any drugs?	Never	Rarely	Sometimes	Often	Very Often	Refused
11a. [If ORB11 = 1,2,3, or 4]. In the <u>past month</u>, did you **switch from injecting** to other ways of using drugs (like snorting, smoking, or swallowing) for 7 days or longer, for any reason?	No	Yes	Refused			
11b. [If ORB11 = 1,2,3, or 4]. In the <u>past month</u>, did you **inject fentanyl** (intentionally or accidentally)?	No	Yes	Not sure	Refused		

12. In the <u>past month</u>, what is the **longest amount of time** (days in a row) that you went without using <u>any drugs</u>?

___ ___ *(xx)* (Number of days, maximum 30) or ☐ Refused

13. In the <u>past month</u>, have you gone 2 or more days without using opioids?

00 = No 01 = Yes 98 = Refused

13a [If Yes to 13]. In the <u>past month</u>, how many times did you **not use opioids for 2 or more days** at any time?

___ ___ *(xx)* (Number of times) or ☐ Refused

13b [If Yes to 13]. After the most recent time that you went without using opioids for 2 days, **how much did you use** when you started using opioids again?

00 = About the same; 01 = More; 02 = Less; 97 = Don't Know; 98 = Refused

<u>SCORING:</u>

Score is sum of ORBC items 1, 2, 3, 4, 5, 6, 7, 8, 9, 10, 11 (44 total points possible)

Harm Reduction Checklist (HRC)

Administered at all study visits by RA

Assessment time frame:
At the baseline visit: Ask the questions about activities in the past three (**3) months.**
Month 1 Study Visit: Ask the questions about activities in the past **month**.
Month 3 Study Visit: Ask the questions about activities in the past **two (2) months**.
Month 6 Study Visit: Ask the questions about activities in the past **three (3) months**.
Month 7 Study Visit: Ask the questions about activities in the past **month**.

STEP 1: Receipt of and Participation in Harm Reduction Services

If "Yes/achieved" is marked at any previous visit, indicate "Yes/achieved" for Question 1 below and skip to Question 2. If all previous visits are "No", ask Question 1.

1. Have you been provided information about safer injection practices and/or needle exchange programs?

☐No
☐Yes/achieved

Indicated at previous visits:

2. **Always ask:** How often have you used safer injection practices (e.g., doing a tester shot, taking turns using, using safer injection spaces/locations)?

☐I don't currently inject drugs
☐Very often/always
☐Often
☐Sometimes
☐Rarely
☐Never

M1, M3, M6, M7:

If "Yes/achieved" is marked at any previous visit, indicate "Yes/achieved" for Question 3 below and skip to Question 3a. If all previous visits are "No", ask Question 3 and 3a.

3. Have you been given a fentanyl test strip?

☐No
☐Yes/achieved

3a. **If "Yes/achieved", always ask**: How often have you used a fentanyl test strip?

☐Very often/always
☐Often
☐Sometimes
☐Rarely
☐Never

Indicated at previous visits:

M1, M3, M6, M7:

If "Yes/achieved" is marked at any previous visit, indicate "Yes/achieved" for Question 4 below and skip to Question 5. If all previous visits are "No", ask Question 4.

4. Have you received information about the risk of overdose with combined use of benzodiazepines (sedatives like Xanax or Ativan) and opioids?

☐No
☐Yes/achieved

Indicated at previous visits:

M1, M3, M6, M7:
If "Yes/achieved" is marked at any previous visit, indicate "Yes/achieved" for Question 5 below and skip to Question 6. If all previous visits are "No", ask Question 5.

5. Have you received information regarding decreased tolerance and increased risk for overdose following a period of not using drugs?
   - ☐No
   - ☐Yes/achieved

   Indicated at previous visits:

M1, M3, M6, M7:
If "Yes/achieved" is marked at any previous visit, indicate "Yes/achieved" for Question 6 below and skip to Question 7. If all previous visits are "No", ask Question 6.

6. Have you received a Narcan kit?
   - ☐No
   - ☐Yes/achieved

   Indicated at previous visits:

7. **Always ask:** If you were to experience an overdose today, would Narcan be immediately available to you or someone in your social/support network to use for you?
   - ☐Very Often/always
   - ☐Often
   - ☐Sometimes
   - ☐Rarely
   - ☐Never

8. **Always ask:** Have you experienced depression, anxiety, or other mental health issues?
   - ☐No
   - ☐Yes

M1, M3, M6, M7:
If "Yes/achieved" is marked at any previous visit, indicate "Yes/achieved" for Question 8a below and skip to Question 8b. If this is the first time 8a is asked or if all previous visits are "No", ask Question 8a.

   8a. If "Yes" to Q8, did you receive information regarding treatment for mental health issues?
   - ☐No
   - ☐Yes/Achieved

   Indicated at previous visits:

M1, M3, M6, M7:
If "Yes/achieved" is marked at any previous visit, indicate "Yes/achieved" for Question 8b below and skip to Question 9. If this is the first time 8b is asked or if all previous visits are "No", ask Question 8b.

   Indicated at previous visits:

   8b. If "Yes" to Q8, did you engage or are you engaging in treatment for mental health issues?
   - ☐No
   - ☐Yes/achieved

9. **Always ask:** Have you experienced serious medical problems such as problems with infections (e.g., skin, lung), liver problems (like hepatitis), breathing?

☐No
☐Yes

9a previous visit: *If "Yes/achieved" is marked at any previous visit, indicate "Yes/achieved" for Question 9a below and skip to Question 9b. If this is the first time 9a is asked or if all previous visits are "No", ask Question 9a.*

9a If "Yes", did you receive information about treatment for your serious medical problems?

☐No
☐Yes/achieved

Indicated at previous visits

9b previous visit: *If "Yes/achieved" is marked at any previous visit, indicate "Yes/achieved" for Question 9b below and proceed to Question 10. If this is the first time 9b is asked or if all previous visits are "No", ask Question 9b.*

9b. If "Yes" to Q9, did you receive or are you receiving treatment for your medical problems?

☐No
☐Yes/achieved

Indicated at previous visits:

10. **Always ask:** Have you been **prescribed** opioid painkillers (e.g., hydrocodone, oxycodone)?
*Please specify that we are asking about **prescribed** medications in this question; illicitly obtained prescription opioids should be captured in the TLFB. Methadone prescribed for opioid use disorder would be captured in medications for OUD questions.*

☐No/achieved
☐Yes

10a. If "Yes", how much do you take per day?

Medication Name	Dose/ Units	Average Number of Doses Per Day
Drop down: Hydrocodone<br>Oxycodone<br>Morphine<br>Hydromorphone<br>Oxymorphone<br>Fentanyl<br>Other<br>If, "Other" specify:	Numeric field (0-999)<br>Drop down: mg, mcg, mg/mL	Numeric field
Drop down: Hydrocodone<br>Oxycodone<br>Morphine<br>Hydromorphone<br>Oxymorphone<br>Fentanyl<br>Other<br>If, "Other" specify:	Numeric field (0-999)<br>Drop down: mg, mcg, mg/mL	Numeric field
Drop down: Hydrocodone<br>Oxycodone<br>Morphine<br>Hydromorphone<br>Oxymorphone<br>Fentanyl	Numeric field (0-999)<br>Drop down: mg, mcg, mg/mL	Numeric field

Other<br>If, "Other" specify:		
Drop down: Hydrocodone<br>Oxycodone<br>Morphine<br>Hydromorphone<br>Oxymorphone<br>Fentanyl<br>Other<br>If, "Other" specify:	Numeric field (0-999)<br>Drop down: mg, mcg, mg/mL	Numeric field
Drop down: Hydrocodone<br>Oxycodone<br>Morphine<br>Hydromorphone<br>Oxymorphone<br>Fentanyl<br>Other<br>If, "Other" specify:	Numeric field (0-999)<br>Drop down: mg, mcg, mg/mL	Numeric field

Scoring Instructions:

Each item will be considered to be "met" based on the following logic:

- Items 1, 3, 4, 5, and 6: Response of "Yes/achieved"
- Item 2: Response of "I don't currently inject drugs" or "Very often/always"; or a value at any visit more frequent than that of Baseline value ("I don't inject drugs response" as the highest value)
- Items 3a and 7: Response of "Very often/always"; or value at any visit more frequent than that of Baseline value
- Item 8: Response of "No" OR "Yes" and 8a and 8b = "Yes" at the same visit
- Item 9: Response of "No" OR "Yes" and 9a and 9b = "Yes" at the same visit
- Item 10: Response of "No/achieved"

A participant will be considered to have achieved Step 1 if

(1) any previously "unmet criteria" becomes met,
(2) total score of "met criteria" **increases** from baseline at any time during study (even if score subsequently decreases),
(3) if item #7 is indicated "Often" OR "Very often/always" at baseline **and** is again indicated as "Often" OR "Very often/always" at any follow-up visit.

These are additional scoring items that can be calculated in the database or calculated by Nick at the end of the study and would be helpful variables for the lead team.

HRC Summary Items:

1. Total Points at this visit (number of checked boxes, out of 14): 0-14

Steps Achieved Assessment (SAA)

RA administered to participant, Steps 2-7

STEP 2

1. BL: In the past month, have you participated or completed any of the following:
   M1, M3, M6, M7: Since your last study visit (*see above)*, have you participated in or completed any of the following:
   a. Scheduled an appointment for substance use or addiction treatment:
      ☐No
      ☐Yes

   b. Attended a 12-step or recovery-oriented meeting (e.g., in-person or on-line; AA, NA, SMART, Celebrate Recovery, In the Rooms):
      ☐No
      ☐Yes
         i. BL: If, "Yes", how many in the past month?
         M1, M3, M6, M7: If, "Yes", how many since the last study visit?
         _ _ meetings (0-500, 0-999)

   c. Attended a session or had an interaction with a peer recovery specialist, either in-person, virtual, text, or phone session:
      ☐No
      ☐Yes
         i. If, "Yes", how many times did you meet? xx_(0-100)
         j. If, "Yes", how did you meet with the peer? *Check all that apply.*
            ☐In-person
            ☐Video chat
            ☐Phone call
            ☐Texting

   d. Attended a formal substance use treatment appointment (virtual or in-person, including medication and/or psychosocial treatment, including outpatient, intensive outpatient, or residential treatment):
      ☐ No
      ☐ Yes
      BL: If, "Yes", how many in the past month?
      M1, M3, M6, M7: If, "Yes", how many since last study visit? XX (0-500, 0-999)

M3, M6, M7: STEP 3

2. M3, M6, M7: In the past 3 months, have you participated in any of the following at least 3 times?

   a. M3, M6, M7: Attended 3 or more in-person or on-line meetings at a 12-step group (e.g., AA, NA, SMART, Celebrate Recovery, In the Rooms)?
      ☐No
      ☐Yes

   b. M3, M6, M7: Met with a peer recovery specialist either in-person, virtual or phone session at least 3 times?
      ☐ No
      ☐Yes
         i. If, "Yes", how did you meet with the peer? *Check all that apply.*
            ☐In-person
            ☐Video chat
            ☐Phone call
            ☐Texting

   c. M3, M6, M7: Attended an in-person or virtual formal SUD treatment appointment (medication and/or psychosocial treatment) at least 3 times?
      ☐No
      ☐Yes

Steps 4-7

RA to Participant: "The following questions have to do with medications for the treatment of opioid use disorder, which include methadone, buprenorphine/Suboxone, or naltrexone/vivitrol."

The RA will then conduct timeline follow-back procedures for MOUD use since the last study visit was completed. The RA will then complete the following questions based on the information from the timeline follow-back.

3. BL, M1, M3, M6, M7: Did the participant take any medication for opioid use disorder (MOUD) in the past month (30 days)?
4. M3, M6, M7: Did the participant take any medication for opioid use disorder (MOUD) in the past three (3) months (90 days)?
5. M6, M7: Did the participant take any medication for opioid use disorder (MOUD) in the past six (6) months (180 days)?

- ☐ No
- ☐ Don't Know
- ☐ Yes

a) BL, M1, M3, M6, M7: How many days out of the past 30 did the participant take medication for opioid use disorder (MOUD)? _______ days (0-30)

b) M3, M6: How many days out of the past 90 did the participant take medication for opioid use disorder (MOUD)? _______ days (30 day answer-90)

c) M6: If "Yes", how many days out of the past 180 did the participant take medication for opioid use disorder (MOUD)? _______ days (90 day answer-180)

6. If "Yes" to any MOUD, which formulation(s) were used: *(check all that apply)*

a. Methadone ☐ No ☐ Yes

i. Max daily dose: (1mg-500mg)

b. Buprenorphine☐ No ☐ Yes

i. If "Buprenorphine", which formulation was used:

☐Under the tongue

☐ Monthly shot

☐ Weekly shot

ii. What was the max dose : ___ ___.___ *(XX.X)* (0.5mg-80mg)

c. Naltrexone (not including Narcan/naloxone given for overdose reversal): ☐ No ☐ Yes

If "naltrexone", which formulation was used:

☐Oral

☐Monthly shot

i. Max oral dose: ___ ___ ___ *(xxx)* (1mg-300mg)

MOUD Confirmation Assessment (MCA)

1. Was self-report of MOUD confirmed via toxicology screen:
   ☐ Yes – buprenorphine and methadone only
   ☐ No – positive self-report for naltrexone
   ☐ No – unable to perform

   a. If "Yes", was the result consistent with the positive MOUD (buprenorphine or methadone) reported via self-report?
      ☐No
      ☐Yes

2. If toxicology screen was inconsistent or not completed, were any secondary methods for confirmation obtained? *(select all that apply)*

   Confirmation with treatment provider: ☐No ☐Yes
   a. If "Yes", specify date provided: __/__/____ *(mm/dd/yyyy)*
   Note: must be within 90 days of study visit

   b. If "No", why? __________________________

   Confirmation with pharmacy of prescription written and/or dispensed consistent with MOUD availability in the timeframe: ☐No ☐Yes
   c. If "Yes", specify date participant was provided prescription bottle: __ __/__ __/__ __ __ __ *(mm/dd/yyyy) Note: must be within 90 days of study visit*
   d. If "No", why? __________________________

   Participant provided prescription bottle at study visit (in-person or virtual); confirmed name, medication name, and date dispensed consistent with MOUD availability in the time frame: ☐No ☐Yes

   e. If "Yes", specify date participant was provided prescription bottle: __ __/__ __/__ __ __ __ *(mm/dd/yyyy) Note: must be within 90 days of study visit*
   f. If "No", why? __________________________

Table S1. Table of study assessments and procedures.

Assessment	Time Needed to Complete	Screening	Random-ization	Baseline	Day 30	Day 90	Day 180	Day 210	As Needed
		(ED Visit/Enrollment) – Virtual or In-person			Phone, Virtual, or In-person	Phone, Virtual, or In-person	Phone, Virtual, or In-person	Phone, Virtual, or In-person	
**Screening**									
Screening Enrollment Form	5 mins	X							
Prisoner Status Assessment	2 mins	X							
Demographics	5 min	X							
Patient Health Questionnaire-9	2 mins	X			X	X	X	X	
Screening Exit Survey	10 mins	X							
Written Informed Consent	30-45 mins	X							
**Inclusion/Randomization and Study Visits**									
Locator Information Form	5-10 mins			X	[X]	[X]	[X]		[X]
Overdose Information	3 mins			X	X	X	X	X	
Urine Drug Test	5 mins			X	X	X	X	X	
Overdose Risk Behavior Checklist	3 mins			X	X	X	X	X	
Harm Reduction Checklist	3 mins			X	X	X	X	X	
Step 2-7 Assessment Form	4 mins			X	X	X	X	X	
MOUD Confirmation Assessment	3 mins				X	X	X	X	
MOUD Current Status	2 mins			X	X	X	X	X	
Readiness Ruler	1 min			X	X	X	X	X	
Alcohol and Substance Use History	10 mins			X					
Timeline Follow-Back (7 day)	3 mins			X	X	X	X	X	
DSM-5 Checklist	5-10 mins			X	X	X	X	X	
ED MOUD Administration	-			X					
Assessment of Recovery Capital	8 mins			X	X	X	X	X	
Chronic Pain History	1 min			X					
Tobacco Use History	2 mins			X					
Fagerstrom Test for Nicotine Dependence	2 mins			X					
Cannabis Use Assessment	1 min			X			X	X	
Quality of Life	3 mins			X	X	X	X	X	
Crime and Criminal Justice	3 mins			X	X	X	X	X	
Treatment Satisfaction Form	5 mins							X	X
**Administration or Non-Visit Based Forms**									
Mental Health Follow-up Assessment	2 mins								X
ED Visits and Hospitalizations	5 mins								X
TAU Characterization Form	15-20 mins								X
**Remote Assessments**									
Mobile Weekly Surveys	3-5 mins				X[b]	X[b]	X[b]	X[b]	
**PILOT Intervention**									
PILOT Intervention Activities Log	1-5 mins			X	X	X	X	X	
Supervisor Log	3-5 mins							X	
Progress Note	2-10 mins							X	

Notes: [b]=administered weekly. [X]= assessment is reviewed and updated as needed
